# Wafer-scale transfer route for top–down III-nitride nanowire LED arrays based on the femtosecond laser lift-off technique

**DOI:** 10.1038/s41378-021-00257-y

**Published:** 2021-04-23

**Authors:** Nursidik Yulianto, Andam Deatama Refino, Alina Syring, Nurhalis Majid, Shinta Mariana, Patrick Schnell, Ruri Agung Wahyuono, Kuwat Triyana, Florian Meierhofer, Winfried Daum, Fatwa F. Abdi, Tobias Voss, Hutomo Suryo Wasisto, Andreas Waag

**Affiliations:** 1 Institute of Semiconductor Technology (IHT), Technische Universität Braunschweig, Hans-Sommer-Straße 66, Braunschweig, 38106 Germany; 2 Laboratory for Emerging Nanometrology (LENA), Technische Universität Braunschweig, Langer Kamp 6, Braunschweig, 38106 Germany; 3 Research Center for Physics, Indonesian Institute of Sciences (LIPI), Jl. Kawasan Puspiptek No. 441-442, Tangerang, Selatan 15314 Indonesia; 4 Engineering Physics Program, Institut Teknologi Sumatera (ITERA), Jl. Terusan Ryacudu, Way Huwi, Lampung Selatan, Lampung 35365 Indonesia; 5 Institute of Energy Research and Physical Technologies, Technische Universität Clausthal, Leibnizstraße 4, Clausthal-Zellerfeld, 38678 Germany; 6 Institute for Solar Fuels, Helmholtz-Zentrum Berlin für Materialien und Energie GmbH, Hahn-Meitner-Platz 1, Berlin, 14109 Germany; 7 Department of Engineering Physics, Institut Teknologi Sepuluh Nopember (ITS), Jl. Arif Rahman Hakim, ITS Campus Sukolilo, Surabaya, 60111 Indonesia; 8 Department of Physics, Faculty of Mathematics and Natural Sciences, Universitas Gadjah Mada, Sekip Utara PO Box BLS 21, Yogyakarta, 55281 Indonesia

**Keywords:** Nanowires, Nanowires, Nanowires, Electronic devices

## Abstract

The integration of gallium nitride (GaN) nanowire light-emitting diodes (nanoLEDs) on flexible substrates offers opportunities for applications beyond rigid solid-state lighting (e.g., for wearable optoelectronics and bendable inorganic displays). Here, we report on a fast physical transfer route based on femtosecond laser lift-off (*fs*-LLO) to realize wafer-scale top–down GaN nanoLED arrays on unconventional platforms. Combined with photolithography and hybrid etching processes, we successfully transferred GaN blue nanoLEDs from a full two-inch sapphire substrate onto a flexible copper (Cu) foil with a high nanowire density (~10^7^ wires/cm^2^), transfer yield (~99.5%), and reproducibility. Various nanoanalytical measurements were conducted to evaluate the performance and limitations of the *fs*-LLO technique as well as to gain insights into physical material properties such as strain relaxation and assess the maturity of the transfer process. This work could enable the easy recycling of native growth substrates and inspire the development of large-scale hybrid GaN nanowire optoelectronic devices by solely employing standard epitaxial LED wafers (i.e., customized LED wafers with additional embedded sacrificial materials and a complicated growth process are not required).

## Graphical abstract

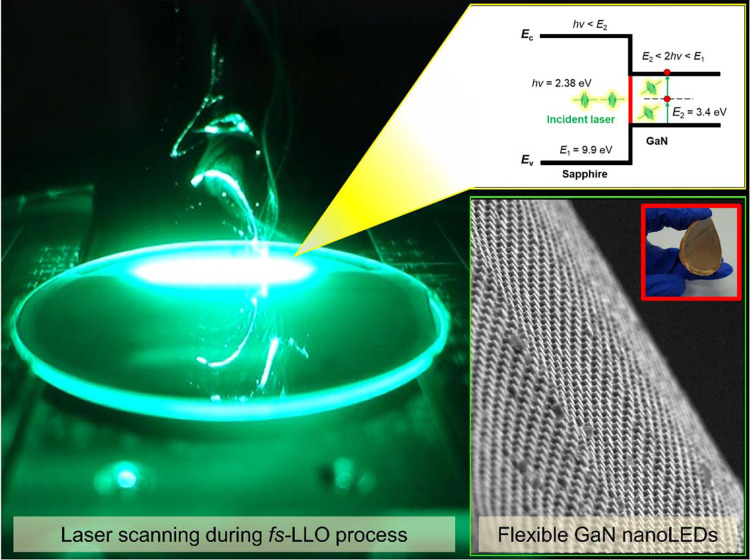

## Introduction

Gallium nitride (GaN) has been continuously used in the light-emitting diode (LED) industry for almost three decades since the introduction of commercial high-brightness GaN blue LEDs in 1993 by Nakamura et al.^1–3^. Their invention has propelled the previously less-considered GaN materials into mainstream optoelectronic research and development. Today, GaN is ubiquitously found in a wide range of device applications beyond solid-state lighting (e.g., electronics, displays, optical microscopy, visible light communications, fuel cells, optogenetics, and biochemical sensors)^3–9^. It is fascinating to witness that the interest in GaN LEDs is now shifting from standard, square millimeter-sized high-power LEDs to micrometer-sized pixels (10–50 µm)^10,11^. Owing to their high brightness, high transparency, long lifetime, low power consumption, and short response time, GaN microLEDs are an ideal candidate for display applications and have the potential to replace liquid crystal display (LCD) and organic LED (OLED) technology^12,13^.

Considering the processing complexity, cost, and performance of these devices, hybrid integration of microLEDs with a transistor backplane is more preferable than a monolithic fabrication of high-electron-mobility transistors and microLEDs on a single wafer. Thus, GaN microLEDs grown on sapphires are commonly flip-chip bonded onto silicon-based complementary metal oxide semiconductor (CMOS) drivers, in which a laser lift-off (LLO) transfer process subsequently releases the native substrate^14,15^. For state-of-the-art LLO-based transfer technology in the LED industry, various short-pulsed laser sources (e.g., excimer and Q-switched nanosecond lasers (355 nm)) have frequently been used, where delamination of the GaN layer from the sapphire occurs based on a direct photon absorption mechanism in the *n*-GaN part located at the GaN/sapphire interface^16^. In fact, this technological concept is mature and generally used in LED fabrication.

The further shrinkage of GaN-based LEDs to submicron sizes (<1 µm) has been demonstrated by forming them as core-shell, disc-in, and dot-in indium gallium nitride (InGaN)/GaN nanowire LEDs (nanoLEDs) in a bottom–up approach; these devices are known as nanoLEDs^11,17–19^. InGaN/GaN nanoLED arrays with tunable peak emission wavelengths in almost the entire visible spectrum (red, green, and blue—RGB) can be obtained on a single wafer solely by varying the wire diameters (200–600 nm)^11^. Furthermore, since the initial demonstrations on self-assembled vertical GaN nanowires in the late 1990s, continuous improvements have been mainly devoted to controlling the bottom–up fabrication of random and aligned nanowires by molecular beam epitaxy and metal-organic chemical vapor deposition^20–22^. However, bottom–up GaN nanowire fabrication has revealed several limitations (i.e., difficult-to-control concentrations of impurities, defects along the wire in the *c*-plane direction, and the formation of inhomogeneous structures). Thus, in most cases, these selectively grown GaN nanowires possess different geometries (i.e., heights, diameters, and shapes), although their growth substrates have been lithographically patterned^23,24^. In contrast, the epitaxial growth of planar GaN film LEDs has reached a high level of maturity. Starting from planar heterostructures, nanoLEDs can also be fabricated by a top–down method, which has lately become more popular, obviating the disadvantages of the bottom–up approach by simply etching the planar thin-film LED into disc-in nanoLEDs^24–26^.

One of the key processes in microdisplay fabrication is the wafer-scale transfer of the micro/nanoLED structures onto another suitable carrier, e.g., a CMOS silicon wafer or a flexible metal foil. Flexible metal foils are particularly useful for wearable optoelectronic devices such as flexible displays and optical smart sensors. Earlier studies have demonstrated the simple integration of core-shell InGaN/GaN nanoLEDs into a flexible substrate by embedding the devices within elastomeric polydimethylsiloxane (PDMS) layer^27–29^. Although several hybrid flexible GaN LEDs emitting blue, green, and white light were successfully fabricated in those reports, their main building blocks (i.e., self-assembled nanoLEDs) possessed random positions, irregular heights, and discordant geometries. Moreover, their wire transfer was still performed manually using a scalpel on a small device area (1 cm × 1.5 cm), leading to a low process reproducibility level^27^. Another conventional nanowire LED transfer method through a pick-and-place process could take several hours in preparation, especially, when electron beam lithography was used to form contacts, causing a limited number of transferred nanowires (low yield) and inefficient integration^30^. Utilizing selective photoelectrochemical (PEC) etching, GaN LED devices could also be separated from their growth substrates. Nonetheless, this method is very sensitive to the doping concentration and requires a customized LED wafer with an additional embedded sacrificial material (e.g., zinc oxide^31^, chromium nitride^32^, boron nitride^33^, or InGaN^34^), which definitely induces higher costs and more difficulties in optimizing heteroepitaxial growth.

**Fig. 1:**
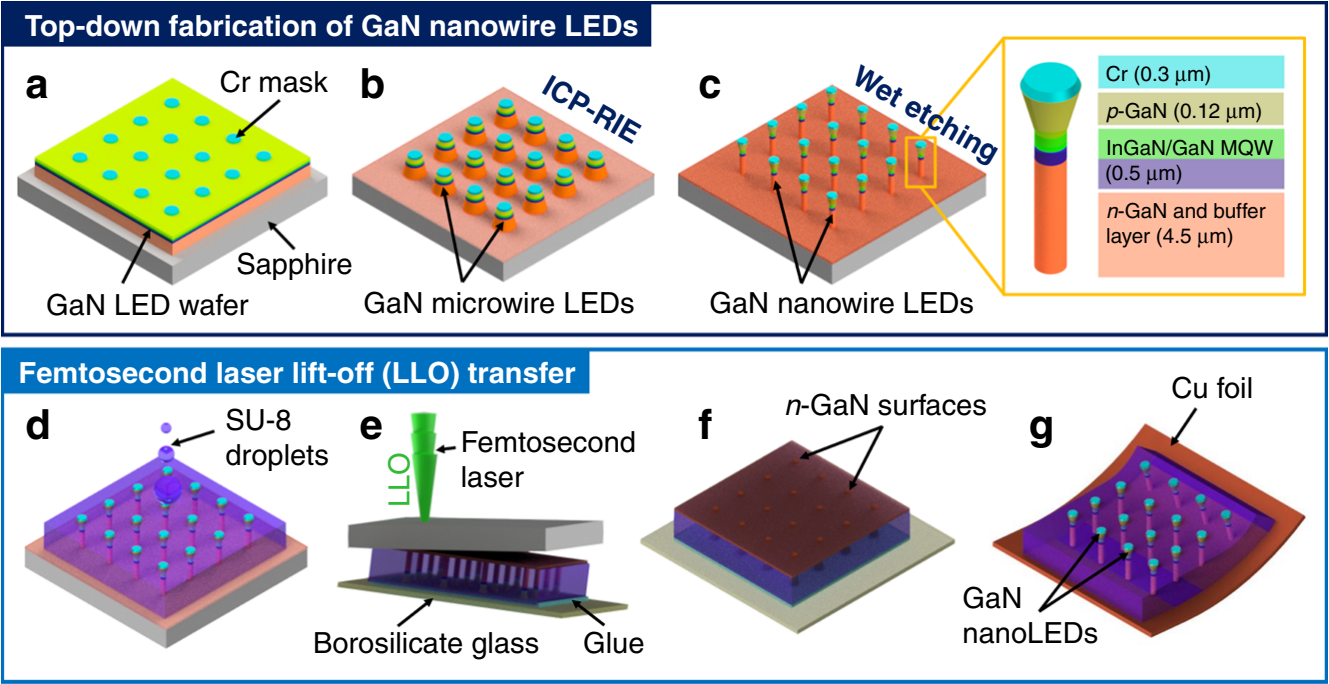
Wafer-scale transfer route for vertical GaN nanowire light-emitting diodes (nanoLEDs) on a nonconventional substrate. **a** The top–down fabrication includes photolithography to pattern chromium (Cr) masks on an LED wafer, **b** inductively coupled plasma reactive ion etching (ICP-RIE) using an SF_6_/H_2_ gas mixture at room temperature, and **c** potassium hydroxide (KOH)-based wet etching. The femtosecond laser lift-off (*fs*-LLO) transfer process consists of **d** polymer spin-coating and back-etching, **e** transfer of the whole GaN nanoLED structure from sapphire onto temporary borosilicate glass substrate and its subsequent delamination from the sapphire using a femtosecond laser, **f** sapphire removal resulting in the opening of an *n*-GaN surface, and **g** cleaning of GaN nanoLEDs attached to a copper (Cu) foil with the freely exposed *p*-GaN layer on the top side

Herein, we employed a fast physical transfer technique based on ultrashort femtosecond laser lift-off (*fs-*LLO) to realize vertical GaN nanoLEDs on nonconventional substrates (see Fig. 1). In our previous reports, *fs*-LLO at a wavelength of 520 nm and a pulse width of 350 fs was used to release GaN LED films with small area sizes (0.5 × 0.5 mm^2^ and 1 × 1 cm^2^) from their original sapphire substrates^35,36^. However, in those studies, the GaN LEDs were either not fully fabricated as a completely functional device in a cleanroom (i.e., *n*-GaN opening was not performed and metal contact was not realized)^35^ or processed into non-nanostructured LED devices^36^. Moreover, although thin-film LEDs could be attached to the Cu foil, their flexibility on a large scale was not demonstrated. Thus, in this work, we investigated the potency of *fs*-LLO to transfer wafer-scale GaN nanoLEDs embedded in SU-8 from a sapphire substrate onto a Cu foil, in which commercial GaN LED wafers were used during laser processing. The greatest advantage of the *fs*-LLO technique is its ability to transfer the semiconductor film with a higher bandgap than the photon energy from the excitation laser source. This method could be applied to separate AlGaN UV LEDs from their sapphire substrates, where multiphoton absorption from a femtosecond laser occurred during the transfer process^36^. Therefore, *fs-*LLO is a versatile technique for creating thin-film UV-visible GaN LEDs. Meanwhile, choosing a femtosecond laser source at visible wavelengths (e.g., 520 nm) offers precise optical alignment during sample preparation. Different laser sources with a longer wavelength of 1030 nm (near-infrared) and a pulse width of 350 fs can also be used to yield multiphoton absorption instead of only two-photon absorption. This technique has been reported to slice GaN thin film from its original bulk substrate (i.e., bulk GaN)^37^.

Furthermore, highly ordered nanowires were fabricated in a top–down approach from full two-inch InGaN/GaN LED wafers combining photolithography, inductively coupled plasma reactive ion etching (ICP-RIE), and wet etching. SU-8 polymer and copper (Cu) foil were used as flexible substrates to keep the nanowire arrays in good order and to act as bottom *n*-contacts, respectively. Compared with other flexible substrates made of polymer materials (e.g., polyethylene terephthalate, PDMS, and Kapton polyimide films)^38^, Cu foil provides higher electrical and thermal conductivities in addition to good flexibility^39^. Moreover, the SU-8 thin layer is a transparent and flexible material that is adaptable to the bending of Cu foil substrates^40,41^. The thickness of SU-8 can be precisely adjusted using oxygen plasma-based dry etching. The material characteristics of the transferred nanoLEDs were investigated to assess the strength and weakness of the *fs-*LLO transfer process. The transferred GaN nanoLEDs address the aforementioned manufacturing challenges because they satisfy the small device dimension requirement, can be separated from their rigid native growth substrates straightforwardly, require the only standard LED wafers, and have a high potential to be employed for flexible display platforms, all achieved with a rapid, nonchemical, and scalable method.

## Results and discussion

### Top–down GaN nanoLEDs

A hybrid etching method comprising sequential SF_6_/H_2_-based ICP-RIE and KOH-based wet etching processes was employed to form vertical GaN nanoLEDs out of ~4.6 µm thick planar GaN LED film grown on a sapphire substrate (Fig. 1a–c). During lithography, 300 nm thick spherical chromium (Cr) islands with a diameter of 1 µm were created from a Cr film that had been previously deposited by electron beam evaporation. A metal-based hard mask of the Cr layer was required instead of a photoresist or nanoimprint resist, which is commonly used in the top–down fabrication of vertical silicon (Si) nanowire arrays^42–45^. In terms of the mechanical properties, GaN has a higher hardness, mechanical stability, and stiffness (i.e., Young’s modulus of 300–350 GPa) than Si^46,47^. Thus, the Cr mask must be sustained during the first deep physical etching of the GaN nanoLEDs down almost to the sapphire substrate surface with an etch depth of ~4.6 µm. It was reported in an ICP-RIE study that a Cr mask had high selectivity (up to 8:1) towards GaN, which led to the feasible realization of a microscale GaN waveguide on Ga_2_O_3_^48^. For the etching selectivity, its value strongly depends on the parameters used, in which for physical etching, as in our case, the main influencing parameters are ICP power, high frequency (HF) power, and plasma density^49^. On the one hand, to support their application as a bendable optoelectronic device, GaN nanoLEDs should be completely separated from each other. On the other hand, when further device transfer steps (Fig. 1d–g) are considered, having a very thin *n*-GaN layer holding all the GaN nanoLEDs is more beneficial for maintaining good vertical alignment during processing. Thus, in our transfer route, depending on the employed ICP-RIE recipe, the GaN nanoLEDs are mechanically supported by a remaining *n*-GaN layer with a thickness of 200–400 nm after hybrid etching.

**Fig. 2:**
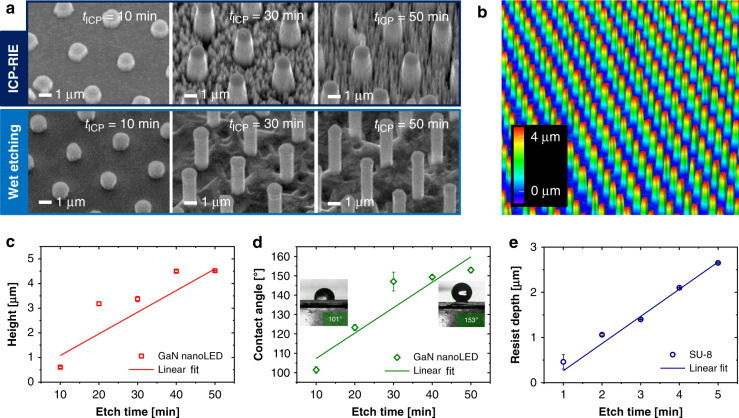
GaN nanoLEDs created by a top–down hybrid etching method (i.e., a process combination of ICP-RIE and wet-chemical etching). **a** Scanning electron microscopy (SEM) images of vertical GaN nanoLEDs processed with different ICP-RIE times (*t*_ICP_ = 10–50 min at room temperature) and constant wet etching durations (20 min at 80°C). **b** 3D contour plot of typical GaN nanoLEDs (*t*_ICP_ = 50 min) measured by confocal laser scanning microscopy (CLSM). **c** Influence of ICP-RIE time on nanoLED height. **d** Water contact angles measured on GaN nanoLEDs with different heights. **e** ICP-RIE duration against the etched SU-8 resist depth

To precisely define the required etching duration during ICP-RIE, we first conducted experiments with dry etching times (*t*_ICP_) varying from 10 to 50 min at 10 min intervals. The other etching parameters were kept constant (i.e., ICP power of 800 W, HF power of 275 W, DC bias of −140 V, pressure of 0.5 Pa, and SF_6_/H_2_ fluxes of 12/100 sccm at room temperature). Fig. 2a depicts the scanning electron microscopy (SEM) images of the GaN nanoLEDs after ICP-RIE (top) and subsequent wet-chemical etching processes (bottom). A longer dry etching duration not only results in higher nanowires but also leads to rougher surfaces that occur on the nanowire sidewalls and the bottom spaces among them. Nonetheless, these rough sidewall surfaces of the GaN nanowires could be smoothed by wet-chemical etching in a potassium hydroxide (KOH)-based etchant at a temperature of 80 °C for 20 min. Thus, the wire diameter also decreased. In case excessive etching time was used, flashlight- or mushroom-like GaN structures would appear instead of GaN cylinders because the wet etching proceeded at a slower rate in both the *p*-GaN and the multiquantum well (MQW) region^50,51^. Fig. 2b shows a confocal laser scanning microscopy image of wafer-scale GaN nanoLED arrays with high homogeneity after dry and wet etching processes. Their typical diameter, height, and pitch are ~700 nm, ~4.5 µm, and 4 µm, respectively.

Figure 2c indicates that for etch times longer than 40 min, the dry etching process in the vertical direction slowed down. The fabricated GaN nanoLEDs have similar heights of (4.50 ± 0.05) and (4.57 ± 0.01) µm when they were etched within 40 and 50 min, respectively. This phenomenon was caused by the higher level of grass-like structures appearing on the bottom GaN surface of the etched area, which prevented further etching (see Fig. 2a and S1a in the Supplementary Information). The occurrence process is very similar to the black silicon phenomenon, where an etching/passivation competition mechanism can lead to the creation of random Si nanospikes with very high aspect ratios in a self-masking way^52,53^. When various carrier substrates (i.e., Si, Ti, Al, and sapphire) were used underneath the GaN films, they could impact the surface profiles of the etched GaN films owing to unwanted contaminants during etching, resulting in different surface roughness levels^54,55^. In our case, running a longer etching process (>50 min) would therefore only further attack the Cr mask and lead to more dominant GaN grasses (Fig. S1a–e in the Supplementary Information). From these experiments, the dry etching rates for GaN and Cr were found to be 900 and 5 nm/min, respectively. In other words, a high selectivity of 180:1 was obtained for Cr as a mask for GaN etching. At *t*_ICP_ = 70 min, the Cr mask was completely stripped off from the GaN nanowire top surface during the ICP-RIE process. Thus, irregular GaN nanorods were yielded since the generated ions in the chamber could directly bombard the GaN top surfaces (Fig. S1c in the Supplementary Information). Afterwards, they were diminished when the sample was chemically etched in a KOH-based solution (Fig. S1e in the Supplementary Information). This method was proven to depend on the target etch depth. A stable hard metal mask that is a few hundred nanometers has to be used during both dry and wet etching processes. The similar obtained nanowire heights for *t*_ICP_ = 20 and 30 min could be due to the variation in chamber condition during the etching process (i.e., the ICP etcher was normally used for structuring not only GaN but also other materials (Si and SU-8 resist)). In addition to being implemented for nanoLEDs, this hybrid technique has been previously employed for building GaN nanowire field-effect transistors with various layer stack compositions (e.g., *n*-GaN and *n-p-n*-GaN)^56–58^.

In terms of the etching plasma used, despite their high toxicity, chlorine (Cl)- or boron (B)-based gases (e.g., BCl_3_/Cl_2_, BCl_3_/SF_6_, N_2_/Cl_2_/O_2_, Cl_2_/Ar/O_2_, Cl_2_/N_2_, and Cl_2_/Ar) were still frequently used for etching GaN structures because of their high selectivity and verticality levels^59–63^. However, as in our case, other plasma sources (e.g., SF_6_/H_2_) can also be used for the dry etching of GaN, although they do not deliver a very smooth surface on the sidewalls due to less chemical selectivity^51,54,64^. For LED devices, however, having smooth sidewalls is a prerequisite before the structures can be further processed. Rough sidewalls with numerous dangling bonds may constitute nonradiative recombination centers, which reduce the photon extraction efficiency^26,65^. Thus, KOH-based etching was carried out to overcome this issue.

Because of its non-centrosymmetric crystal structure, the wurtzite GaN crystal has a polar axis along the (0001) direction^66^. Although the N-polar direction possesses a structure with a single nitrogen bond oriented towards the surface, the Ga-polar direction comprises three nitrogen bonds oriented towards the surface. The N- and Ga-polar GaN materials demonstrated different etching behaviors when they were treated in KOH solution interacting with hydroxide ions (OH^–^)^67–69^. To guarantee the GaN etching occurrence, the hydroxide ions need to have access to the Ga atom. Although hexagonal GaN pyramids can be formed in the N-polar region during etching, Ga-polar areas will remain unetched and perfectly intact^67^. During the initial GaN nanowire etching, Ga atoms are attacked and dissolved by (OH^–^), forming gallium oxide (Ga_2_O_3_) and ammonia (NH_3_). The vertical nonpolar sidewalls have a high density of nitrogen dangling bonds, which are negatively charged and can electrostatically repel the hydroxide ions, thus protecting the gallium back bonds and controlling the etching process^70^. However, if numerous defects exist in the GaN material, such as those found in our microwires after ICP-RIE, a higher number of dangling or defective Ga bonds can promote local etch pit formation, which then allows the hydroxide ions to reach Ga atoms, progressing to the wet etching process. We note that the presence of a top Cr mask is a critical prerequisite to obtain smooth *a*-plane sidewalls. This was proven in our previous report, where GaN nanowires with free *c*-plane (without Cr mask) were wet etched in AZ400K, resulting in irregular cross-section profiles and *m*-plane sidewalls^51^. Indeed, several factors may influence the wet etching rate (e.g., KOH concentration, temperature, etching time, mask material, and solution agitation). In our case, the lateral etching rate of GaN nanowires is typically in the range of 15–20 nm/min^26^.

Although this work has focused on the investigation of the feasibility of the *fs*-LLO-based transfer technique for large-scale GaN nanowire arrays, we additionally conducted wettability experiments on etched GaN nanoLEDs to predict their behavior when interacting with water droplets. This is critical if such platforms will be used as PEC sensors involving the photoluminescence (PL) detection technique^71^. For flexible GaN nanowire optoelectronic devices, silver (Ag) nanowire networks or graphene materials that are diluted in water are often drop-cast or spin-coated as transparent contact electrodes instead of thin indium tin oxide (ITO) films^72–74^. Fig. 2d shows that by keeping the same pitch of 4 µm and increasing the height of GaN nanowires from 1 to 4.6 µm, the measured water contact angle was changed from 101° to 153° (i.e., becoming more hydrophobic). Retaining hydrophobicity levels (>150°) that are too high will not be beneficial for dropping or spin-coating the water-based Ag nanowires, as the droplets will be strongly repelled by the GaN nanowires, leading to ineffective material contact deposition. However, a hydrophilic surface with a contact angle of <90° is also unwanted, as the deposited Ag nanowires will not be distributed homogenously over the wafer^75^. Thus, for optimal wettability conditions, a range between 90° and 150° is suggested.

In addition to wetting behavior, another important parameter is the etching rate of the SU-8 resist, which was used here as a mechanical support layer in addition to the remaining thin *n*-GaN film during the transfer process of the GaN nanoLEDs from the sapphire substrate to the Cu foil (Fig. 1d–g). This embedding polymer could also be used as an insulating layer to avoid a short circuit between *n*-GaN and *p*-GaN layers during *p*-contact creation^27^. The initial thickness of the spin-coated SU-8 was ~5 µm, which completely covered the GaN nanoLEDs (Fig. S2a, b in the Supplementary Information). An etch rate of 600 nm/min was found for the hardened SU-8 resist (see Fig. 2e) when it was etched in an ICP machine using SF_6_/O_2_ plasma. This value is comparable with that in another SU-8 etching study employing the same plasma, in which etching rates of up to 800 nm/min could be obtained with low roughness (i.e., a root mean square surface roughness of < 750 nm) and high anisotropy^76^. Therefore, to reopen the top surface of the GaN nanoLEDs (Fig. 1e and S2c in the Supplementary Information), the filling SU-8 layer needs to be back-etched for 1–2 min depending on its original thickness.

### *fs*-LLO transfer

After the GaN nanoLEDs had been prepared and filled with SU-8 resist, they needed to be temporarily bonded onto borosilicate glass as an intermediate carrier during the *fs*-LLO process. A thin Crystalbond glue layer heated at a temperature of 120 °C was employed to realize the attachment forming the sapphire/GaN nanoLED/adhesive/borosilicate glass stack (Fig. 1e). This thermoplastic adhesive enables simple fast bonding and the removal of parts by thermal cycling. Thus, it has been widely used in the processing of several optoelectronic devices, including flexible optical neural probes embedded with GaN microLEDs^77^.

**Fig. 3:**
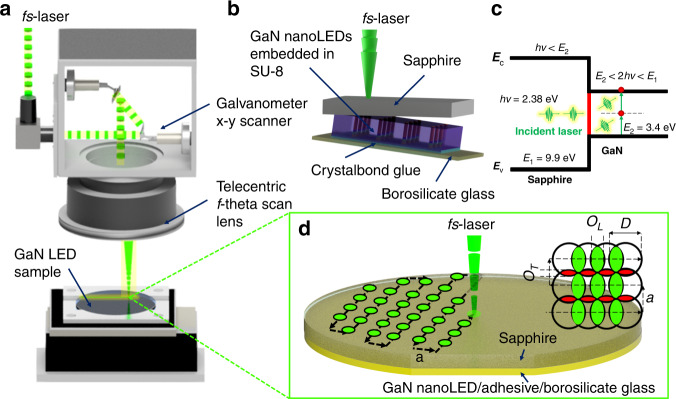
Laser system configuration and micromachining pattern of *fs*-LLO for GaN nanoLEDs. **a** Laser beam path in the setup during the *fs-*LLO process based on raster scanning utilizing a Galvano *x-y* scanner. **b** Schematic of *fs*-LLO for GaN nanoLEDs embedded in an SU-8 layer. The laser light is transmitted through the wafer backside (sapphire). **c** Energy band diagram of *fs*-LLO illustrating two-photon absorption in GaN material using femtosecond pulses at 520 nm. The excitation of two photons (2 *hv*) from the laser possesses higher energy than GaN (*E*_2_) but lower energy than the sapphire (*E*_1_) bandgap. **d** Scheme of the *fs*-LLO pattern showing pulse overlap and number. *O*_*L*_ and *O*_*T*_ are the percentage amounts of overlap between the diameters of two consecutive pulses in the lateral and transverse directions, respectively. *D* and *a* are assigned to diameter of craters in the *n*-GaN layer caused by the beam spot on the GaN surface and the track displacement (transverse pitch between passes), respectively

A laser source with a peak wavelength (*λ*) of 520 nm, a pulse width (*τ*) of 350 fs, and a repetition rate (*f*) of 200 kHz was used. The *fs*-LLO process was conducted by raster scanning of the femtosecond laser beam across the backside of the sapphire substrate (see Fig. 3a–d). Sapphire has a large energy bandgap (*E*_1_ = 9.9 eV) and is transparent with respect to the laser wavelength. Therefore, photons emitted from the laser source are transmitted through the sapphire and subsequently reach the GaN/sapphire interface. Here, although the excitation laser photons possess lower energy (*hv* = 2.38 eV) than the GaN bandgap (*E*_2_ = 3.4 eV), their high intensity leads to multiphoton absorption in the GaN region near the GaN/sapphire interface, as shown in Fig. 3c. This process is made possible by two-photon absorption^35^. To obtain a homogenous morphology while the laser beam hit the GaN surface, the *fs-*LLO procedure was performed with a laser scanning speed (*ν*) of 2 m/s, a scan pitch or distance between two pulses in the transverse direction (*a*) of 10 µm, and a beam width (*D*) of ~20 μm at the laser focus position on the target surface (see Fig. 3d). Thus, the working distance around the focal region is ~0 mm from the surface of the sample^35^, whereas the distance between the sample surface and the telecentric lens is ~12 cm. Using Eq. (S3.1) and (S3.2) in the Supplementary Information, the degree of overlap between the diameters of two consecutive pulses in the lateral (*O*_*L*_) and transverse (*O*_*T*_) directions was determined to be ~50%. Moreover, the pulse number per area (*n*/*A*) can be calculated using Eq. (S3.3), resulting in one pulse for every 100 µm^2^. The laser pulse energy (*E*_*p*_) was kept constant at ~3.3 µJ per pulse, corresponding to a laser energy density or peak fluence (*Φ*_0_) of ~1.5 J/cm² at the sample plane. In our previous report, this pulse energy level was proven to be able to yield a high lift-off success rate of up to ~70%^35^. The integrated fluence (*Φ*_int_) calculated from Eq. (S3.4) amounts to 3.3 J/cm^2^ (Fig. S3a, b in the Supplementary Information).

**Fig. 4:**
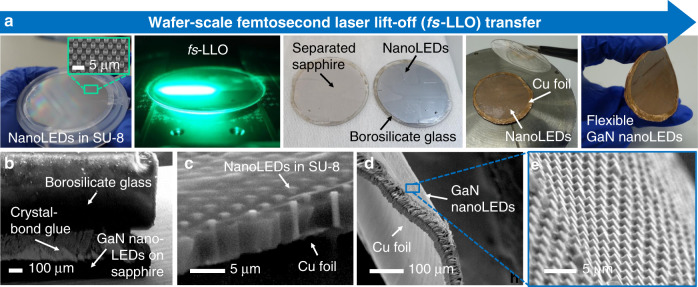
GaN nanoLEDs in the entire process chain of the wafer-scale *fs*-LLO transfer route starting from their top–down patterning to hybrid integration onto a Cu foil. **a** Photographs of the wafer-scale *fs*-LLO transfer route for creating flexible GaN nanoLEDs: spin-coating of SU-8 on a nanopatterned LED wafer, etching of SU-8 by an SF_6_/O_2_-based ICP-RIE process exposing the *p*-GaN surface, *fs-*LLO processing, the release of nanoLEDs from a sapphire substrate, attachment of nanoLEDs onto Cu foil, and acetone cleaning (left-to-right). **b** GaN nanoLEDs on sapphire after being attached to a temporary borosilicate glass substrate. **c** Released GaN nanoLEDs after *fs*-LLO and attachment to Cu foil. The nanoLED arrays were embedded in an SU-8 membrane and still fully covered with Crystalbond glue. **d**–**e** GaN nanoLEDs that were embedded in a SU-8 membrane and attached to a flexible Cu foil after the cleaning procedure

The conditions of the InGaN/GaN nanoLEDs in the entire process chain of the wafer-scale *fs*-LLO transfer route starting from their top–down patterning to hybrid integration onto a Cu foil are presented in Fig. 4a and Video S1 in the Supplementary Material. Prior to *fs*-LLO, the top–down patterned GaN nanoLEDs were filled with SU-8 resist followed by a hardening process. Then, they were tied to a temporary transfer substrate of 500 µm thick borosilicate glass using Crystalbond adhesive at a temperature of 120 °C, in which a vertical force of 1 N was applied during the bonding procedure (Fig. 4b). By multiphoton absorption during the *fs*-LLO process, GaN was decomposed into metallic Ga and gaseous N_2_ over the whole wafer area. The presence of residual metallic Ga covering the *n*-GaN surface of the released sample was apparent from the metallic reflectivity^78^. Although N_2_ was released from the sample to the air, the oxidation of Ga in ambient O_2_ resulted in a Ga_2_O_3_ layer on the surface^79^. To remove the Ga debris and Ga_2_O_3_ layer, the sample was cleaned using a mixed solution of (1:1) hydrochloric acid (HCl) and deionized water (DI H_2_O) for 2 min at room temperature^80^. This cleaning process can be accelerated using boiled HCl^81,82^. The lifted-off GaN nanoLEDs were then attached to a Cu foil. As a result, the stacking of borosilicate glass/Crystalbond glue/GaN nanoLEDs on the SU-8/Cu film could be realized. Removal of the borosilicate glass was performed at a temperature of 120 °C when the crystalbond adhesive was simultaneously melted. To completely strip off the adhesive residues, the integrated LED stack was cleaned with acetone and isopropanol. Once the temporary borosilicate glass substrate had been pulled away from the LED chips, a wafer-scale GaN nanoLED arrays on Cu foil could be yielded (Fig. 4c–e). It should be noted that for further *p*-contact creation, an ICP-RIE process using SF_6_/O_2_ gases for 1–2 min was required. Hence, the top parts of GaN nanowires that were previously embedded in the SU-8 membrane could be freely exposed (Fig. 4e).

To estimate the transfer success rate of this *fs*-LLO for GaN nanoLEDs, the surface conditions of a full two-inch released sapphire wafer were investigated after release using an optical microscope (see Fig. S4a–e in the Supplementary Information). We employed the image analysis program ImageJ to calculate the areas containing defects (nonlifted GaN nanoLEDs). The transfer failures were identified from the dark areas in the captured images. Although the homogeneity of optoelectrical characteristics of the GaN nanoLEDs was not tested in this case because the *p*-contact over the whole wafer surfaces had not been created and optimized (i.e., the investigation was limited to the physical appearance of the transferred nanowires on the borosilicate glass and Cu foil, as depicted by Fig. S4f, g in the Supplementary Information), a transfer success rate of ~99.5% could be obtained with a density of 2.5 × 10^7^ wires/cm^2^. In addition, SEM images were also taken at several different positions from the wafer-scale GaN nanoLEDs on Cu foil (see Fig. S4h–k in the Supplementary Information). Their large homogenous distribution over the carrier substrate can be confirmed by means of the captured nanoLED arrays located at the edge and middle areas. Small cracks of the SU-8 and *n*-GaN supporting layers were also found after device bending.

### NanoLED material characteristics

**Fig. 5:**
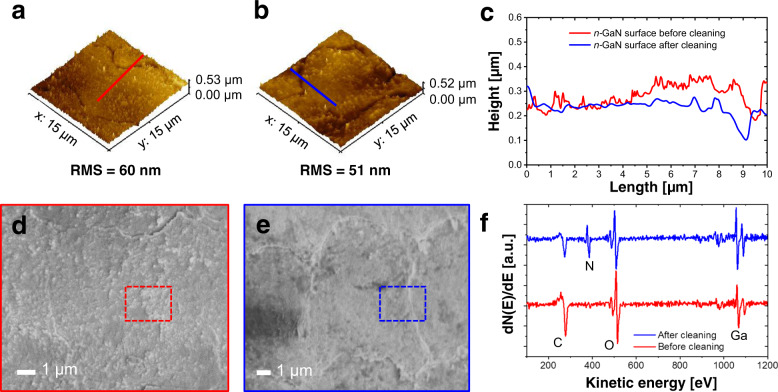
Properties of the *n*-GaN surface resulting from *fs*-LLO. Atomic force microscopy (AFM) height profile images of *fs*-laser-irradiated *n*-GaN surfaces **a** before and **b** after hydrochloric acid (HCl) cleaning. **c** Cross-sectional profiles of both samples showing different surface roughness conditions. SEM images of craters on the released *n*-GaN layer **d** before and **e** after HCl etching. **f** Auger electron spectra of *n*-GaN surfaces obtained from the *fs-*LLO process before and after HCl treatment

Several methods for material characterization were applied to obtain insights into the relevant processes involved in the GaN nanoLED transfer route. First, we studied the effect of HCl-based cleaning on the GaN surface morphology and composition after *fs*-LLO using atomic force microscopy (AFM) and Auger electron spectroscopy (AES). This chemical treatment can be employed to remove part of the Ga debris on the surface of the detached *n*-GaN layer, which may hinder the formation of good contact for the final device^81,82^ From the AFM measurements, root mean square roughness values of 60 and 51 nm were determined for a laser-irradiated, detached *n*-GaN surface before and after HCl cleaning, respectively (see Fig. 5a–c). This shows that this chemical etching could assist in the formation of smoother GaN surfaces. This argument is also supported by the SEM images showing a large part of a GaN crater (see Fig. 5d) and the overlapping region between two adjacent craters (see Fig. 5e), indicating that protrusions on the surfaces were diminished after the sample had been dipped in a diluted HCl solution. Nonetheless, the pattern of the laser beam with a scan pitch of 10 µm could still be observed, with maximum crater depths of ~15 and ~10 nm before and after HCl etching, respectively.

In addition to their topography, the released *n-*GaN surfaces were characterized by AES to analyze their elemental composition (see Fig. 5f). Prior to HCl cleaning, the surface region of the as-lifted sample contained ~31.9% O and 44.5% C, and the Ga-to-N concentration ratio was 6.4. Applying HCl etching reduced both the O and C contents to 26.5% and 25.4%, respectively. The Ga-to-N ratio dropped to a value of 1.7, suggesting that in addition to most of the carbon contamination, the Ga debris on the surface was largely etched away. The residual oxygen AES signal originates at least partly from the native surface oxidation of GaN to Ga_2_O_3_ in the air after etching. From another study related to the fabrication of 3D microchannels in bulk GaN by wet-chemical-assisted *fs*-laser ablation, it was experimentally demonstrated using X-ray photoelectron spectroscopy (XPS) that the chemical composition of the GaN surfaces was altered when they were cleaned using HCl solution^78^. The Ga-to-N concentration ratios were reduced from 1.82 to 1.67 when the *fs*-laser-irradiated GaN surfaces had been treated in the HCl etching process. This reduced value was attributed to the removal of a Ga-rich layer or debris on the ablated surfaces. In addition to HCl solution, other chemicals (e.g., ammonium hydroxide (NH_4_OH), ammonium sulfide ((NH_4_)_2_S), hydrofluoric acid (HF), KOH, and buffered oxide etch solutions) have been applied to remove contaminants, including native surface oxides on GaN surfaces. In these studies, AFM, AES, and XPS have been commonly utilized to compare the results among different cleaning techniques^83–85^. Solutions of HCl, HF, and NH_4_OH were found to be effective for oxide removal^80^. It was also suggested that the cleaning process can be more effective when annealing or heating is involved. Using thermal desorption at a temperature of >900 °C, effective removal of all contaminant species (e.g., carbon contaminants) on the GaN surface was demonstrated for HCl-treated samples^80,83^.

**Fig. 6:**
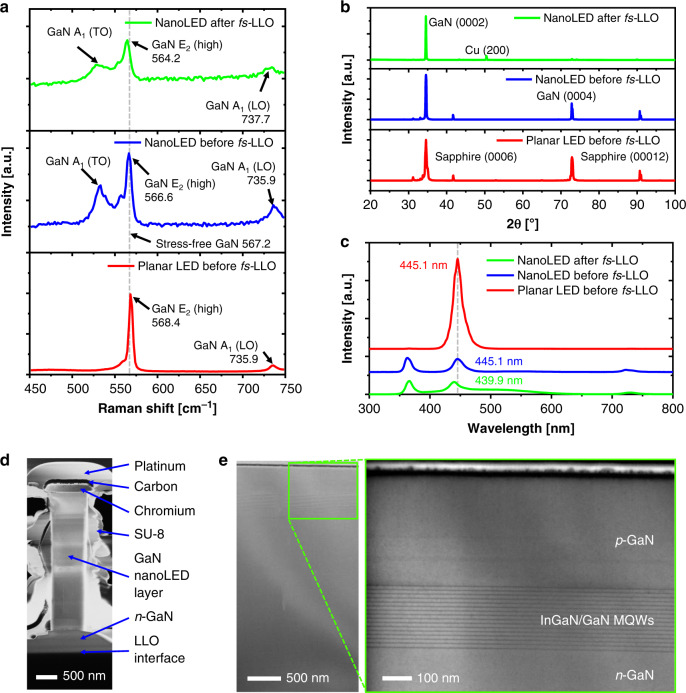
Material characteristics of GaN planar LED and nanoLEDs before and after *fs*-LLO. **a** Raman, **b** X-ray diffraction (XRD), and **c** photoluminescence spectra of the InGaN/GaN nanoLEDs before and after *fs*-LLO in comparison with those of the InGaN/GaN planar LED. All measurements were carried out at room temperature. Cross-sectional focus-ion beam (FIB)-SEM images of **d** single GaN nanoLEDs and **e** thin-film LEDs released by *fs-*LLO. A thin platinum-on-carbon layer was deposited on the LED top surface during sample preparation. The 16-fold InGaN/GaN multiquantum wells (MQWs) were still intact after laser irradiation

As both nanostructuring and *fs*-LLO processing could potentially affect the crystalline and optical properties of the initially metal-organic vapor-phase epitaxy (MOVPE)-grown LED stack, further analysis of three different samples (i.e., planar LED and nanoLEDs before and after *fs*-LLO) at different stages of the wafer processing route was carried out. Figure 6a compares the Raman spectra of the pristine planar LED with those of the nanoLED before and after *fs*-LLO. All samples were examined in the backscattering direction with the excitation laser beam perpendicularly impinging on the wafer surface (along the *c-*axis of the GaN nanoLED crystal stack). The laser excitation energy (2.38 eV) is below the bandgap energies of the InGaN/GaN MQWs (2.79 eV) and GaN (3.39 eV), assuring that the Raman spectra purely originate in a non-resonance regime^86^. All three samples feature the E_2_ (high) and A_1_ (LO) modes of hexagonal wurtzite GaN^87^. Transversal optical (TO) phonon modes (e.g., A_1_ (TO) mode) remain silent for the analysis of GaN in the *c*-axis orientation and thus are not obtained in the present backscattering configuration of the Raman setup^86,88,89^. In contrast, the E_2_ (high) mode is very sensitive to strain and has been commonly employed to quantitatively determine the stress (*σ*) in GaN epilayers^90–92^. Here, unstressed GaN is described with E_2_ (high) at 567.2 cm^−1^ (dashed line in Fig. 6a), whereas compressive/tensile stress results in a shifted E_2_ (high) Raman peak^88,91^. The E_2_ (high) modes of the planar LED and the nanoLEDs before and after *fs*-LLO are detected at 568.4, 566.6, and 564.2 cm^−1^, respectively (see Fig. S5a in the Supplementary Information). Furthermore, the stress of each sample is estimated by using the following expression:

1 $$\sigma = \frac{{\Delta \omega }}{k}$$

with the difference between the measured and unstrained E_2_ (high) Raman modes (Δ*ω* = 567.2 cm^−1^−E_2_ (high)) and a constant Raman stress coefficient (*k* = 4.3 cm^−1^ GPa^−1^)^91^. Using Eq. (1), the planar GaN epilayer results in a compressive stress value of *σ* = −0.279 GPa, whereas the nanoLED before *fs*-LLO reveals a nearly stress-free value of *σ* = 0.139 GPa. This result suggests that the originally compressively stressed InGaN/GaN LED epilayers are relaxed through transformation into nanowires by the hybrid etching (ICP-RIE and KOH etching) process with sufficiently high aspect ratios and free surface areas. After conducting *fs*-LLO and subsequent transfer onto the foreign Cu substrate, the E_2_ (high) mode shifts even further towards lower wavenumbers and thus results in well-pronounced tensile stress of *σ* = 0.698 GPa. From other reports, such tensile stress normally occurred within the GaN LEDs that had been transferred from sapphire onto unconventional flexible substrates (e.g., polyimide and plastic)^92,93^. For example, Horng et al.^92^ demonstrated that shifts of the E_2_ (high) Raman mode are more pronounced for strongly bent samples. Furthermore, the GaN A_1_ (TO) peak was observed at 533.2 cm^−1^ after the patterning of the nanoLED and the *fs-*LLO process (Fig. 6a, blue and green lines), which might be because during Raman measurement, the laser was also scattered by planes other than the *c*-plane on GaN nanowire surfaces^94,95^.

X-ray diffraction (XRD) 2*θ* scans of the as-grown planar LED, on-wafer nanoLED, and *fs*-LLO-transferred nanoLED samples were employed to assess potential changes in the GaN crystal quality that occurred during the nanopatterning and transfer processes. In the X-ray diffractograms of nanoLEDs before and after *fs*-LLO, the (0002) main peak of GaN slightly shifted from 34.56° to a lower diffraction angle to 34.54°, whereas the full width at half maximum values remained constant at 0.28° (Fig. 6b). This indicates that the GaN crystal did not degrade substantially after being transferred onto Cu foil^96^. However, the intensity ratio between the (0004) and (0002) peaks of GaN decreased, and their satellite peaks were reduced after *fs*-LLO (Fig. S5b in the Supplementary Information). A similar intensity ratio change was observed in GaN samples etched in sulfuric/phosphoric acid solutions^97^, in which samples with larger strains seem to have a smaller (0004)/(0002) ratio. This suggests that the *fs*-LLO process introduces additional tensile strain, which agrees with the Raman spectroscopy results, as discussed above. Moreover, a peak of (00012) sapphire with a very low intensity was apparent, indicating that slight sapphire debris was attached to the released GaN during the transfer process (see Fig. S5c in the Supplementary Information).

PL spectra (excitation energy of 3.81 eV) for all three samples (i.e., as-grown unpatterned InGaN/GaN LED material, as-etched nanoLEDs, and as-*fs*-LLO-released nanoLEDs) are shown in Fig. 6c. The planar LED revealed a very pronounced PL peak at ~445 nm attributed to the recombination of electron-hole pairs in the InGaN/GaN MQWs. In contrast, the PL emission of the GaN signal (at ~363–366 nm) was barely visible in the planar LED because most of the excitation laser was absorbed inside the upper regions of the LED stack. After nanowire structuring by ICP-RIE, the underlying GaN buffer layer was directly exposed to the PL laser beam. Consequently, this led to an intensified PL signal of the GaN peak at ~363 nm. Although the peak attributed to the MQW slightly decreased, which could be explained by a reduction in the active emission area inside the excitation volume, the PL peak wavelength remained at 445.1 nm, suggesting no severe differences in the InGaN/GaN layers between the planar and nanoLEDs. However, more pronounced changes in the peak positions were found after *fs*-LLO. In particular, a broader PL signal at a wavelength of ~489 nm was discovered that was ascribed to the underlying Cu substrate (Fig. S5d in the Supplementary Information). The nanoLED after *fs*-LLO also exhibited a redshift of the GaN peak to ~366 nm, which could be explained by an expansion of the crystal lattice that qualitatively agrees with the increase in tensile stress obtained from the Raman analysis (cf. Fig. 6a). Although this is a reasonably consistent finding, it should be noted that the spatial resolutions of the micro-PL and micro-Raman systems are not identical, which limits further quantitative correlations at this point. Finally, a blueshift of the InGaN/GaN peak to 439.9 nm was observed, indicating a similar trend to that reported for planar structures after conventional LLO. This could be ascribed to segregation and subsequent reduction of In content in the MQW^98^ or a weakening of the piezoelectric field that originated from a relaxation of the compressive stress and a consequent band flattening through which the overlap area of the electron and hole wave functions increased (which is equivalent to weakening the quantum-confined Stark effect)^90^.

Furthermore, to identify the crystal quality and to confirm the complete stacking structure of the released GaN nanoLED after the *fs*-LLO process, cross-sectional images were obtained using focus-ion beam milling combined with SEM, as displayed in Fig. 6d. Here, the GaN nanoLED was still supported by the 500 nm thick *n*-GaN film underneath. In addition, we also explored thin-film GaN LEDs processed by identical *fs*-LLO recipes for comparison purposes (see Fig. 6e). Although little laser-induced damage was revealed at the bottom part of *n*-GaN, which had a direct interface with the sapphire substrate prior to *fs*-LLO, the LED layer structure including the 16-fold InGaN/GaN MQW layers was intact, and no cracks were found. The depth of these superficial damages caused by laser absorption was ~1–2 µm from the LLO interface (see Fig. 6e). This result is similar to those yielded in other reports from conventional LLO cases^99^, in which the severity and location of structural defects or damage depend on several factors (e.g., applied laser pulse width, wavelength, and GaN absorption coefficient). From those studies, the frequency-tripled Nd:YAG laser (355 nm) and the KrF pulsed excimer laser (248 nm) possessed GaN absorption coefficient values of 6 × 10^4^ cm^−1^ and 2 × 10^5^ cm^−1^, respectively^99,100^. Furthermore, a femtosecond laser with an energy range of 0.80–2.25 eV (wavelength of 1550–550 nm) was reported to result in two-photon absorption coefficient values on GaN between 1 × 10^−11^ and 2.9 × 10^−11^ m/W^101^. However, for our case, a more detailed study related to the GaN absorption coefficient and damage mechanisms caused by *fs*-LLO using a 520 nm wavelength still needs to be performed. In addition to the structural and optical characterizations discussed, the transferred nanoLEDs will have to be further investigated in terms of their final processing steps (e.g., *p*-contact creation) and optoelectrical measurements (e.g., cathodoluminescence tests) to better understand the *fs*-LLO mechanism and to further assess the potential of this technique as a versatile and fast method to realize large-scale GaN nanoLED-based flexible devices.

## Conclusions

A scalable approach to fabricate and transfer top–down III-nitride nanoLEDs from sapphire onto unconventional substrates has been developed. This approach relies on hybrid etching (i.e., a combination of ICP-RIE and KOH-based etching processes), *fs*-LLO, and thermal adhesive bonding. This method has been demonstrated using full two-inch GaN wafers with a transfer yield of up to ~99.5% by maintaining the vertical alignment of the nanoLEDs. To optimize the process in view of future nanoLED production, the hybrid dry–wet etching time was well controlled. After being irradiated with ultrashort laser pulses, the released GaN bottom surface was treated in HCl cleaning solution to remove all the Ga debris resulting from laser irradiation and Ga surface oxide (Ga_2_O_3_). Based on all the measured structural and optical characteristics of the released GaN nanoLEDs on Cu foil, we conclude that their performance and quality can be comparable, according to the measured parameters, with those of original as-grown planar LEDs on sapphire. The compressive stress can even be reduced after nanowire formation, resulting in more relaxed InGaN/GaN MQWs. Despite requiring further processing and characterization steps to realize a fully functioning LED device (i.e., creation of *p*-contact, electroluminescence test, and quantum efficiency analysis), *fs*-LLO has shown promising results as an effective nanoLED transfer route for next-generation high-throughput flexible display assembly.

## Materials and methods

### InGaN/GaN LED wafers

Planar InGaN/GaN blue LED wafers were acquired from the epitaxy competence center (ec^2^) at Technische Universität Braunschweig, Germany and E-Wave Corporation, the United Kingdom (UK). The *fs*-LLO, AFM, Raman spectroscopy, XRD, PL, and AES measurements were performed with E-Wave LED wafers, and the other experimental results presented in this report were achieved with LED wafers from ec^2^. For the samples produced in ec^2^, the MOVPE method was used to grow the LED layers on a two-inch *c*-plane (0001) double-side polished (DSP) sapphire substrate with a thickness of 430 µm inside an Aixtron G3 reactor. Transparent DSP substrates were required to enable the transmission of light from the *fs-*laser during the lift-off process. The epitaxial LED layer stack with a total thickness of ~5 µm consisted of a 2.4 µm thick *n*-GaN buffer layer with a dopant concentration of 9 × 10^18^ cm^−3^, a 2.1 µm thick *n*-GaN layer with a dopant concentration of 2 × 10^19^ cm^−3^, a fourfold multiquantum well (MQW, 13.6 nm period) with InGaN/GaN layers (2.5–3.0 nm and 10.5–11.0 nm), and a 120 nm thick *p*-GaN layer. Commercial two-inch epitaxial InGaN/GaN LED wafers with a center wavelength of 455 nm and DSP sapphire substrates were purchased from E-Wave Corporation, UK. From their provided data, the total thickness of the whole LED layer stack is ~4 µm. A single LED wafer consists of a 3.4 µm thick *n*-GaN layer with a dopant concentration of ~1.5 × 10^19^ cm^−3^, a 0.4 µm thick active layer having 16 pairs of InGaN/GaN MQWs, and a 0.3 µm thick *p*-GaN layer with a dopant concentration of ~8.9 × 10^18^ cm^−3^.

### Top–down GaN nanoLED fabrication

A process combination of photolithography, ICP-RIE, and wet etching was employed to fabricate top–down GaN nanoLEDs. First, a planar GaN LED wafer was cleaned in a boiled mixture of 30% H_2_O_2_ and 98% H_2_SO_4_ (1:1) for 5 min, followed by a dip in 6.5% buffered HF to eliminate undesired organic contaminants. An MJB4 mask aligner (SÜSS MicroTec SE, Germany) was used to perform optical lithography, resulting in circular Cr etch masks with a thickness of 300 nm and a diameter of 1 µm. Prior to the creation of those patterns, a 300 nm thick Cr layer was deposited by the electron beam evaporation method, which was then followed by a chemical lift-off process. The first physical etching (ICP-RIE) of GaN films by an SI 500 C plasma dry etcher (SENTECH Instruments GmbH, Germany) was conducted to define the height of vertically standing nanoLEDs. Here, a typical dry etching recipe was used for our structures (i.e., ICP power of 800 W, HF power of 275 W, pressure of 0.5 Pa, SF_6_/H_2_ fluxes of 12 sccm and 100 sccm at room temperature). To smoothen the rough sidewall surfaces of the GaN nanowires and simultaneously reduce their diameters, the sample was etched using a KOH-based wet-chemical etchant for 20 min at a temperature of 80 °C.

After the GaN nanoLED arrays had been prepared, they were spin-coated using SU-8 resist with a spin speed of 4000 rpm for 35 s and subsequently heated at 95 °C for 45 s. The SU-8 coating membrane possessed a thickness of ~5 µm filling the gaps and covered the nanowires. After the nanowires had been fully covered by SU-8, ICP-RIE was carried out to etch back the embedded SU-8 layer on the sample for 1 – 2 min (i.e., ICP power of 1000 W, HF power of 15 W, SF_6_/O_2_ fluxes of 5/49.5 sccm, pressure of 0.4 Pa, and temperature of 23 °C). Hence, the top surface of *p*-GaN could finally be freely exposed, while the MQW and *n*-GaN layers were still embedded in the SU-8 membrane. The GaN nanoLEDs on sapphire were then temporarily bonded onto a borosilicate glass carrier utilizing thermal glue of Crystalbond 509 (Plano GmbH, Germany) with a melting point at 120 °C. Afterwards, the layer stack was left at room temperature for at least 5 min. Hence, the glue could be hardened, yielding sapphire/GaN nanoLEDs on SU-8/Crystalbond glue/borosilicate glass layers. After releasing the nanoLEDs from the sapphire in the *fs*-LLO transfer process, GaN nanoLEDs on SU-8/Crystalbond glue/borosilicate glass films were attached onto a sticky Cu foil, where their bonding became stronger than that of Crystalbond adhesive at a temperature of 120 °C. Hence, the borosilicate glass could easily be separated (see Video S1 in the Supplementary Material). As a result, the GaN nanoLEDs in the SU-8 membrane were finally fixed onto the Cu foil, where a subsequent cleaning procedure with acetone and isopropanol was also conducted to remove the residues of Crystalbond glue. To prove the functionality of the GaN nanoLED device on Cu foil after *fs-*LLO, an electroluminescence test was conducted using a semiconductor characterization system (4200-SCS Keithley, Keithley Instruments GmbH), as shown in Fig. S6a in the Supplementary Information. It was obvious that blue light was emitted from the GaN nanoLED, although its emission was localized only in a certain area where the microneedle probe was applied on the *p*-GaN contact of the GaN nanoLED. It should be noted that in this study, we have not yet optimized both *p*- and *n*-contacts (i.e., silver nanowires or ITOs were not used in this sample). The I-V characteristics of a single *fs*-LLO-processed GaN nanoLED during electroluminescence tests are depicted in Fig. S6b in the Supplementary Information.

### Laser micromachining setup

In the *fs*-LLO transfer experiments, a Yb-based commercial *fs*-laser source (SPIRIT-1040, Newport Spectra-Physics GmbH, Germany) with a peak wavelength of 520 nm, a pulse width of 350 fs, and a repetition rate of 200 kHz was used. The emitted laser beam passed through a galvanometer scanner (IntelliSCAN III, SCANLAB GmbH, Germany) approaching a telecentric *f*-theta objective, which was then directed to a sample positioner. The mirrors inside the galvanometer scanner allowed fast x-y beam scanning along the sample surface with a velocity of up to 3 m/s, where the incident angle was kept nearly orthogonal to the surface by the telecentric objective. A further detailed description of the *fs*-LLO setup can be found in our previous report^35^.

### Material characterization

To investigate the crystal quality of GaN nanoLEDs before and after *fs*-LLO, XRD measurements were carried out using a Bruker D8 Advance with Cu Kα radiation (*λ* = 0.154 nm), an acceleration voltage of 40 kV, current of 40 mA, and Bragg-Brentano (*θ*–2*θ*) configuration. The PL characterization of GaN nanoLEDs was conducted under continuous-wave excitation (Kimmon, HeCd laser, *λ*_ex_ = 325 nm). The PL was collected with an objective and imaged onto the entrance slit of a spectrometer. Raman spectra were captured on a Renishaw inVia Qontor Raman microscope equipped with an excitation wavelength of 532 nm and a ×100 zoom lens. The GaN nanoLED cross-section was investigated using a Thermo Fisher Helios 5 UX Dual Beam microscope.

The AES spectra of GaN surfaces were recorded using an Omicron NanoSAM Auger electron microscope equipped with a Zeiss Supra field-emission scanning electron microscope (FE-SEM) and a hemispherical electron energy analyzer. The measurements were performed in constant retard ratio scan mode employing a 5 kV electron beam for the excitation source with a tilt angle of 30° to the sample normal position. The AES spectra were acquired on 4 × 4 μm^2^ selected areas. To observe the surface morphology and roughness of the fabricated GaN nanoLEDs, SEM (Leica Cambridge S360FE) and AFM in tapping mode (Veeco Dimension 3100) were employed. The AFM surface scans were measured on an area of 15 × 15 μm^2^ in tapping mode using a silicon microcantilever. All these characterizations were carried out at room temperature.

## Supplementary information

Supplementary Information

Video S1

## Data Availability

All data generated or analyzed during this study are included in this published article (and its Supplementary Information and Material files).
