# Supplementary material for: Wafer-scale transfer route for top–down III-nitride nanowire LED arrays based on the femtosecond laser lift-off technique
Source: Microsyst Nanoeng. 2021 Apr 23;7:32. doi: 10.1038/s41378-021-00257-y (PMC8433433; doi:10.1038/s41378-021-00257-y)
Supplement: Supplementary file 1 — Supplementary Information [file 41378_2021_257_MOESM1_ESM.docx]

Supplementary Information

Wafer-scale transfer route for top-down III-nitride nanowire LED arrays based on the femtosecond laser lift-off technique

**Nursidik Yulianto^1,2,3,*^, Andam Deatama Refino^1,2,4^, Alina Syring^1,2^, Nurhalis Majid^3,5^, Shinta Mariana^1,2^, Patrick Schnell^6^, Ruri Agung Wahyuono^7^, Kuwat Triyana^8^, Florian Meierhofer^1,2^, Winfried Daum^5^, Fatwa F. Abdi^6^, Tobias Voss^1,2^, Hutomo Suryo Wasisto^1,2,*^, Andreas Waag^1,2^**

^1^ Institute of Semiconductor Technology (IHT), Technische Universität Braunschweig, Hans-Sommer-Straße 66, Braunschweig 38106, Germany

^2^ Laboratory for Emerging Nanometrology (LENA), Technische Universität Braunschweig, Langer Kamp 6, Braunschweig 38106, Germany

^3^ Research Centre for Physics, Indonesian Institute of Sciences (LIPI), Jl. Kawasan Puspiptek No. 441-442, Tangerang Selatan 15314, Indonesia

^4^ Engineering Physics Program, Institut Teknologi Sumatera (ITERA), Jl. Terusan Ryacudu, Way Huwi, Lampung Selatan, Lampung 35365, Indonesia

^5^ Institute of Energy Research and Physical Technologies, Technische Universität Clausthal, Leibnizstraße 4, Clausthal-Zellerfeld 38678, Germany

^6^ Institute for Solar Fuels, Helmholtz-Zentrum Berlin für Materialien und Energie GmbH, Hahn-Meitner-Platz 1, Berlin 14109, Germany

^7^ Department of Engineering Physics, Institut Teknologi Sepuluh Nopember (ITS), Jl. Arif Rahman Hakim, ITS Campus Sukolilo, Surabaya 60111, Indonesia

^8^ Department of Physics, Faculty of Mathematics and Natural Sciences, Universitas Gadjah Mada, Sekip Utara PO Box BLS 21, Yogyakarta 55281, Indonesia

* Corresponding authors: Nursidik Yulianto (e-mail: [n.yulianto@tu-braunschweig.de](mailto:n.yulianto@tu-braunschweig.de)) and Hutomo Suryo Wasisto (e-mail: [h.wasisto@tu-braunschweig.de](mailto:h.wasisto@tu-braunschweig.de)).

Contents

[1. Top-down etching of GaN nanowire LEDs S-2](#_Toc66135653)

[2. SU-8 resist membrane creation S-3](#_Toc66135654)

[3. Number of laser pulses on GaN film S-4](#_Toc66135655)

[4. Femtosecond laser lift-off transfer yield S-5](#_Toc66135656)

[5. Material properties S-7](#_Toc66135657)

[6. Electroluminescence characterization S-8](#_Toc66135658)

# Top-down etching of GaN nanowire LEDs


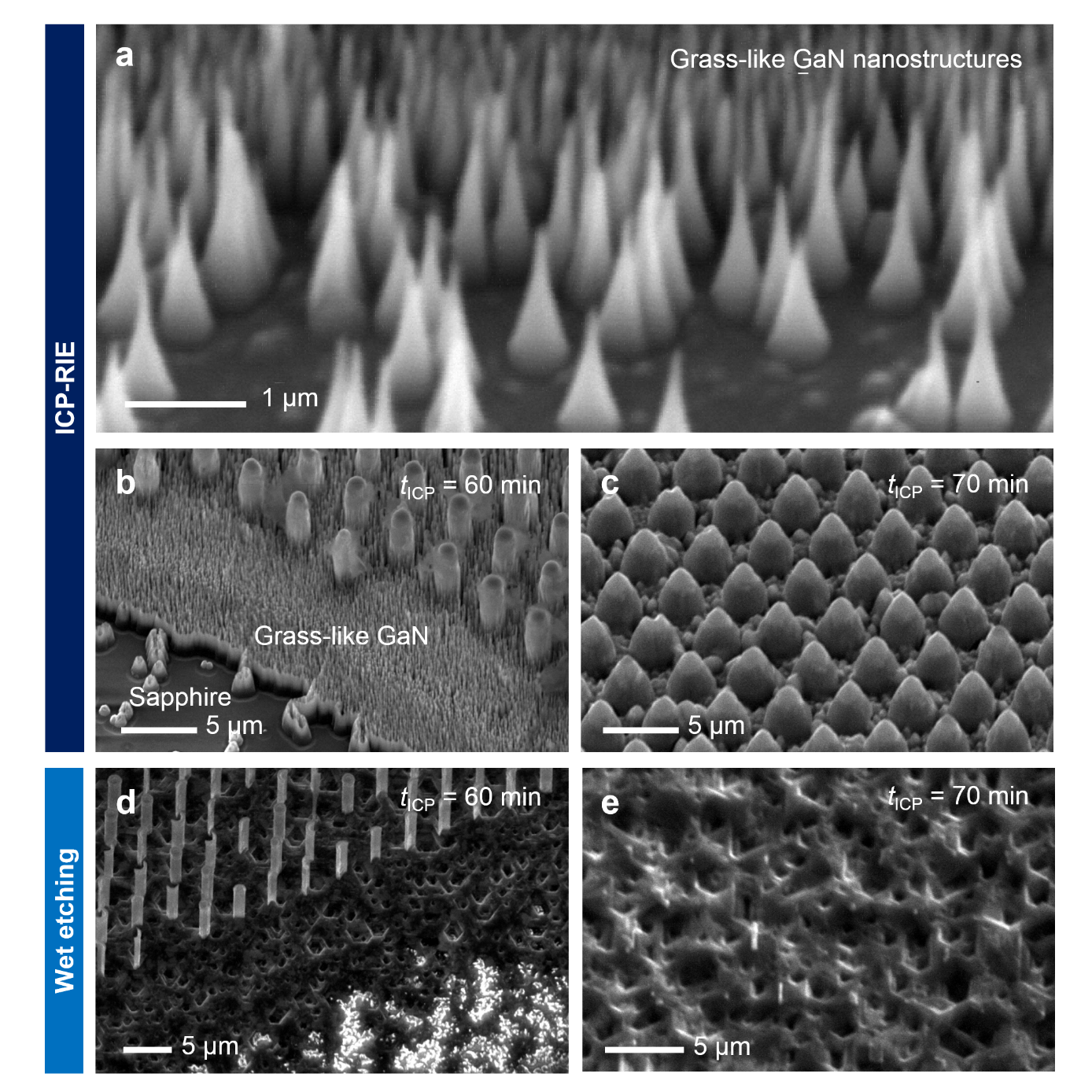


**Figure S1** Top-down etching of GaN nanowire LEDs. **a** Grass-like GaN nanostructures obtained after inductively coupled plasma reactive ion etching (ICP-RIE) process using SF_6_/H_2_ gases. GaN nanoLEDs processed with different ICP-RIE times (**b** **and** **d**: *t*_ICP_ = 60 min; **c** **and** **e**: *t*_ICP_ = 70 min) at room temperature and constant wet etching duration (20 min) at 80°C.

# SU-8 resist membrane creation


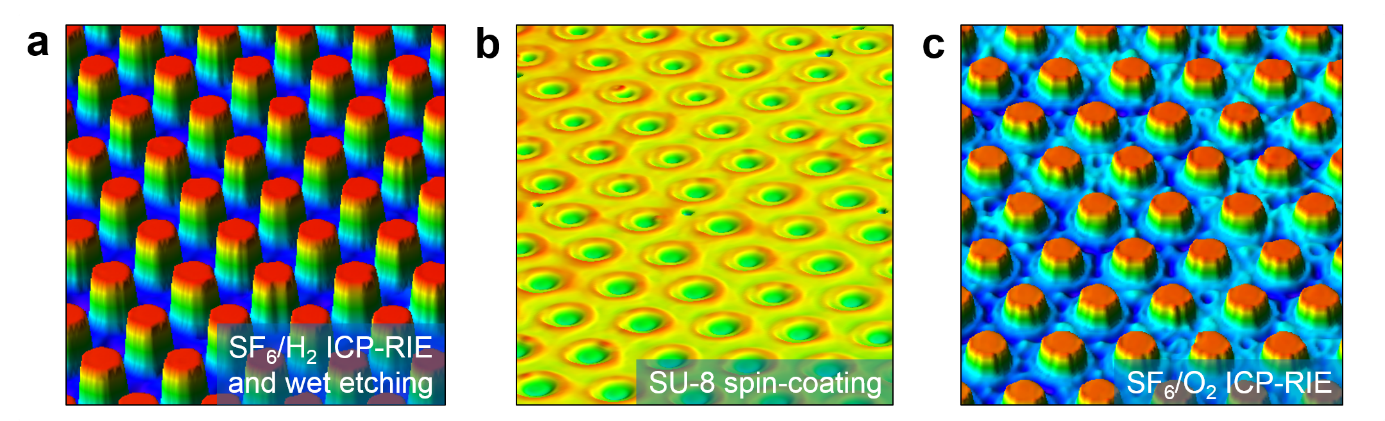


**Figure S2** SU-8 resist membrane creation. Confocal laser scanning microscopy (CLSM) images of GaN nanoLEDs after **a** SF_6_/H_2_-based ICP-RIE, **b** SU-8 filling, and **c** SF_6_/O_2_-based ICP-RIE (back etching). The GaN nanoLEDs could possess freely exposed top surfaces.

# Number of laser pulses on GaN film


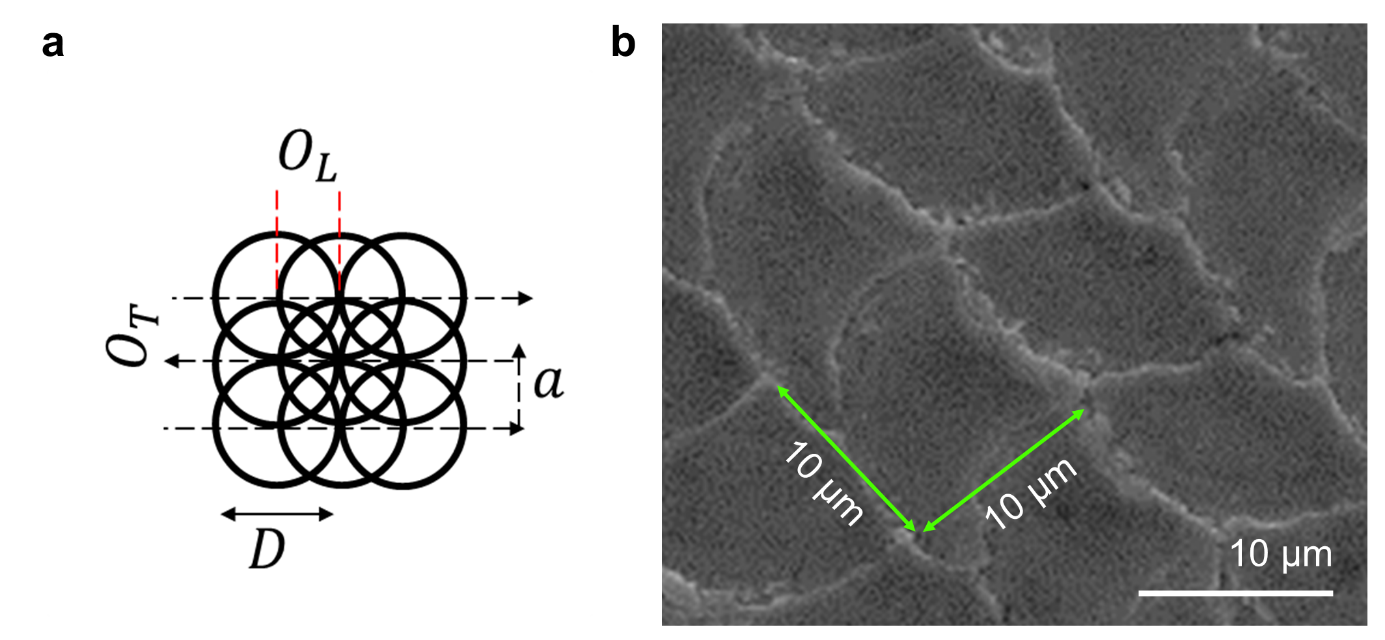


**Figure S3** Number of laser pulses on GaN film. **a** Laser scanning pattern showing overlap and number of pulses for the proposed *fs*-LLO. **b** SEM image of GaN film (*n*-GaN surface) after *fs*-LLO process showing the crater shape and number of pulses per area.

To calculate the degree of overlap (%) between the diameters of two consecutive pulses in lateral $O_{L}$and transverse $O_{T}$directions, the following equations are employed:^36^

$O_{L}= \left( 1-\frac{v/f}{D} \right)\times100\%$ … (S3.1)

$O_{T}= \left( 1-\frac{a}{D} \right)\times100\%$ … (S3.2)

where, $D$,$a$, $f$, and$v$ represent diameter crater that is caused by the beam spot on GaN surface, track displacement (transverse pitch between passes), pulse frequency, and scanning speed of the laser beam, respectively.

The integrated fluence ($Ф_{int}$) value is influenced by pulse energy $(E_{p})$and the number of pulses per area ($n/A$). Therefore, $n/A$ and $Ф_{int}$are calculated by:

$n/A= \frac{1}{a}\times\frac{f}{v}$ … (S3.3)

$Ф_{int}=E_{p}\times n/A$ … (S3.4)

# Femtosecond laser lift-off transfer yield


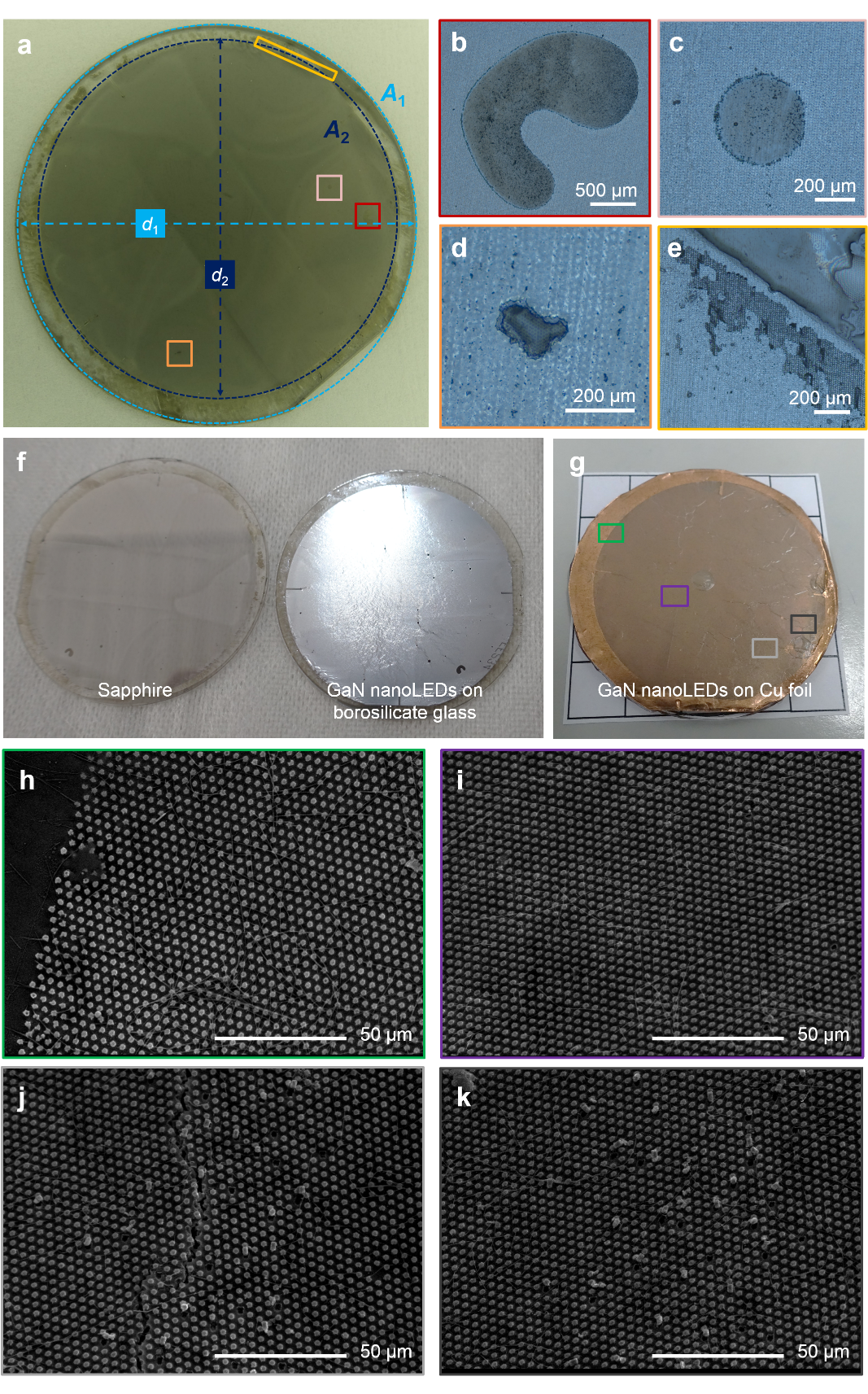


**Figure S4** Transfer yield of *fs*-LLO process. **a** Two-inch released sapphire wafer after *fs*-LLO process containing **b-e** few defects (i.e., non-lifted GaN nanoLEDs). The conditions of GaN nanoLEDs during transfer process on **f** borosilicate glass (intermediate substrate) and **g** Cu foil (final carrier substrate). SEM images taken from different positions in **Figure S4g** showing the GaN nanoLEDs at **h** edge and **i** middle areas. **j** Fractures occurring within SU-8 and *n*-GaN supporting layers after bending. **k** A few slipped nanoLEDs because of transfer process imperfection.

The transfer yield of GaN nanoLEDs can be calculated from **Figures S4 a-e** as follows:

**Total active area (Figure S4a)**

*d*_1_ = 5.08 cm; total wafer area *A*_1_ = 20.26 cm^2^

*d*_2_ = 4.5 cm; total active area *A*_2_ = 15.90 cm^2^

**Total area of non-lifted GaN nanoLEDs**

The defects found in the transferred device might originate from several factors:

- Imperfect transfer process or bubble during stacking between GaN nanoLEDs and borosilicate glass (**Figure S4b and c**) = 0.007 + 0.0003 cm^2^ = 0.0073 cm^2^
- Chemical lift off process (**Figure S4d**) = 0.003 cm^2^
- Photolithography (**Figure S4e**) = 0.07 cm^2^

Total dark (failed) area in **Figures S4b-e** = 0.007 + 0.0003 + 0.003 + 0.07 cm^2^ = 0.0803 cm^2^

**Transfer yield of *fs*-LLO**

Transfer yield = ((total active area – total dark area) / total active area) × 100% = 99.49%

# Material properties


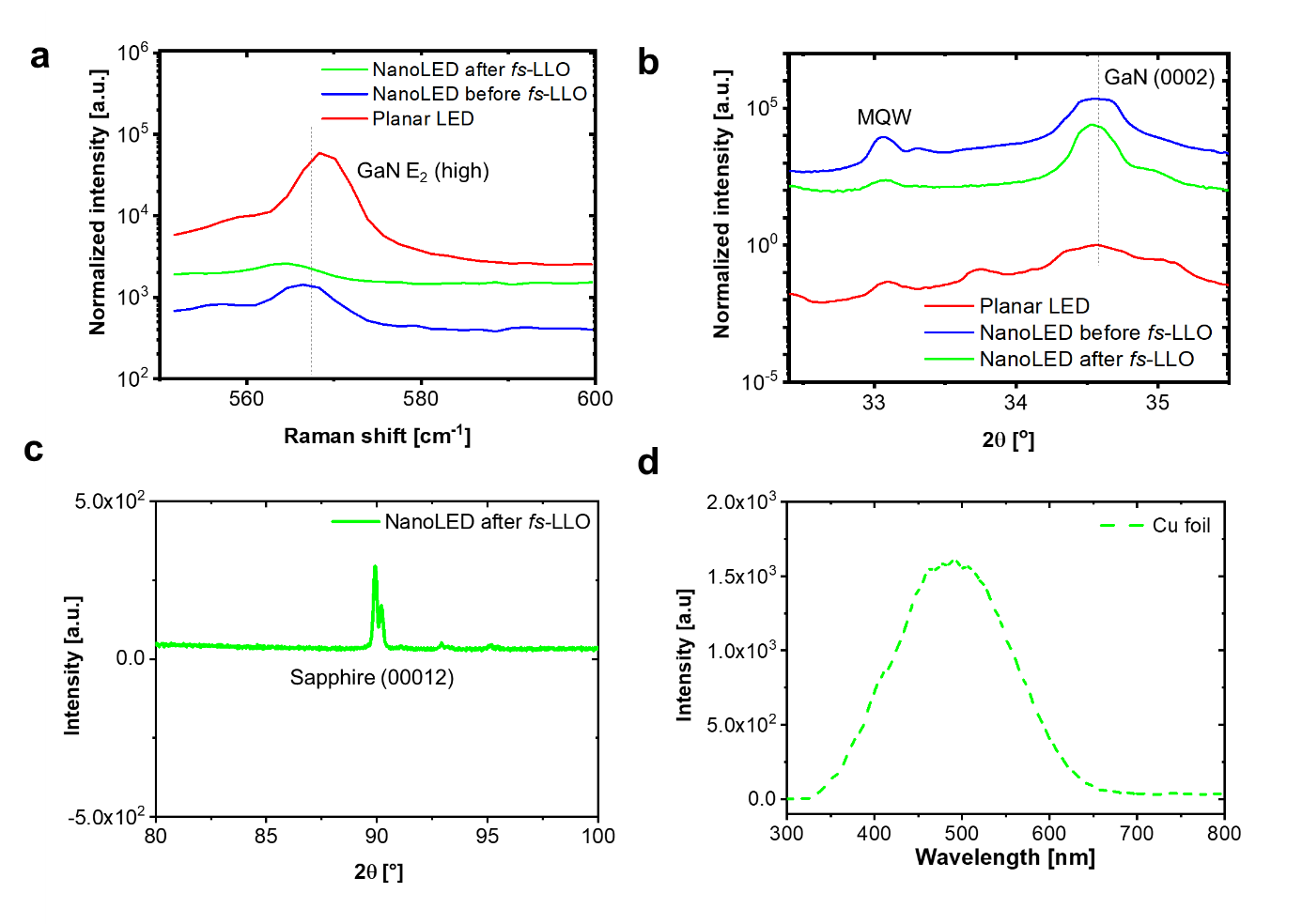


**Figure S5** Material properties of GaN nanoLEDs. **a** Raman spectra of GaN nanoLEDs and planar LED showing shift in E_2_ (high) peak. **b** XRD spectra of GaN nanoLEDs and planar LED exhibiting slight shift in GaN (0002) peak. **c** XRD spectrum of sapphire (00012) debris on the released *n*-GaN surface. **d** Photoluminescence spectrum of Cu foil.

# Electroluminescence characterization


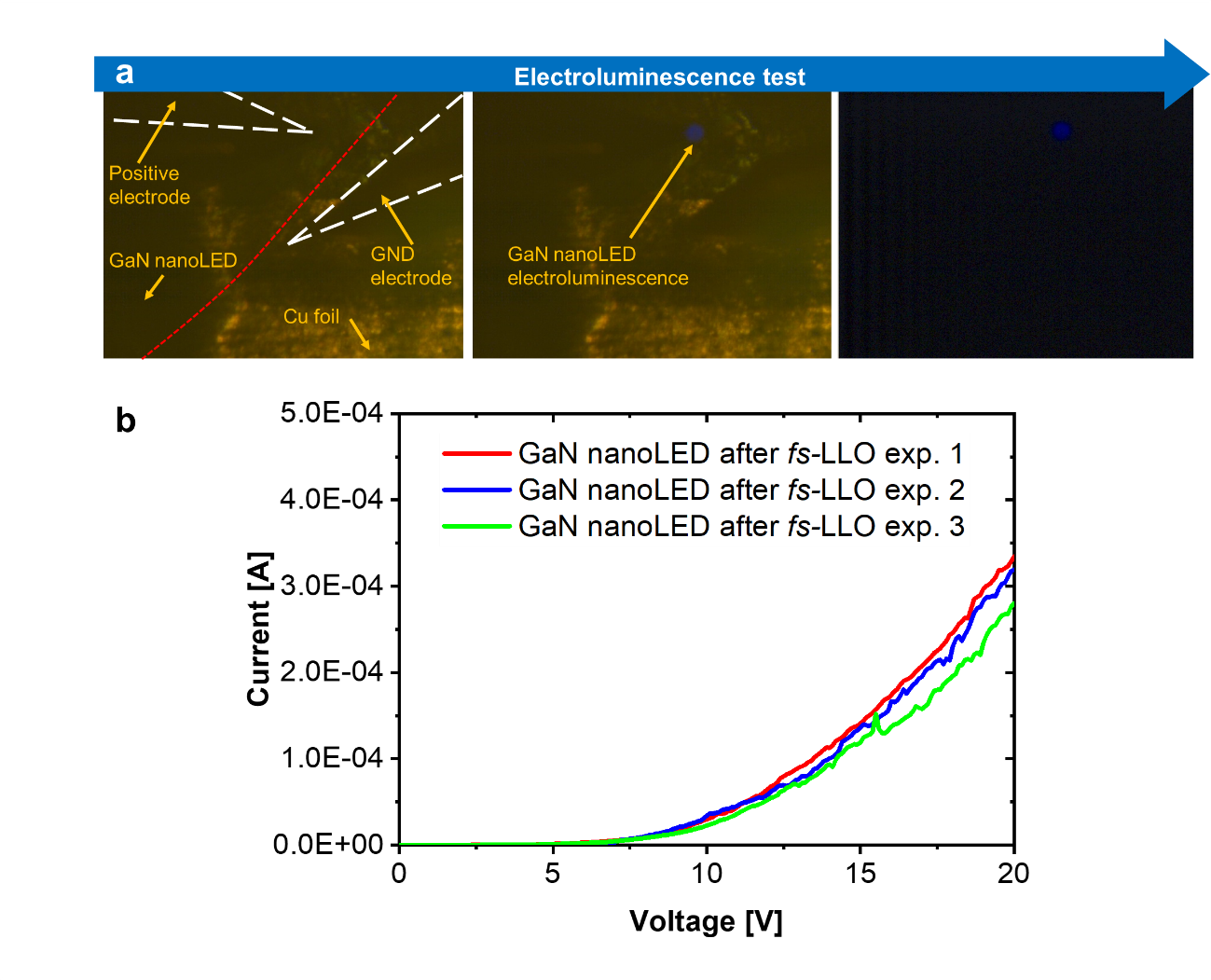


**Figure S6** Electroluminescence characterization. **a** Electroluminescence test results of GaN nanoLEDs attached on a Cu foil after *fs*-LLO process using microprobe tips and a semiconductor characterization system (4200-SCS Keithley, Keithley Instruments GmbH). The blue light emission of active area was localized only in the area where microneedle tip was attached on *p*-GaN contact. Both *p*- and *n*-contacts had not been optimized in this case (i.e., silver nanowires or indium tin oxides were not used in this sample). **b** I-V characteristics of a single *fs*-LLO-processed GaN nanoLED from three repeated measurements.
